# Live Imaging of Micro-Wettability Experiments Performed for Low-Permeability Oil Reservoirs

**DOI:** 10.1038/s41598-017-04239-x

**Published:** 2017-06-28

**Authors:** Hanford J. Deglint, Christopher R. Clarkson, Chris DeBuhr, Amin Ghanizadeh

**Affiliations:** Department of Geoscience, University of Calgary, 2500 University Drive NW, Calgary, Alberta T2N 1N4 Canada

## Abstract

Low-permeability (unconventional) hydrocarbon reservoirs exhibit a complex nanopore structure and micro (µm) -scale variability in composition which control fluid distribution, displacement and transport processes. Conventional methods for characterizing fluid-rock interaction are however typically performed at a macro (mm) -scale on rock sample surfaces. In this work, innovative methods for the quantification of micro-scale variations in wettability and fluid distribution in a low-permeability oil reservoir was enabled by using an environmental scanning electron microscope. Live imaging of controlled water condensation/evaporation experiments allowed micro-droplet contact angles to be evaluated, while imaging combined with x-ray mapping of cryogenically frozen samples facilitated the evaluation of oil and water micro-droplet contact angles after successive fluid injection. For the first time, live imaging of fluids injected through a micro-injection system has enabled quantification of sessile and dynamic micro-droplet contact angles. Application of these combined methods has revealed dramatic spatial changes in fluid contact angles at the micro-scale, calling into question the applicability of macro-scale observations of fluid-rock interaction.

## Introduction

Exploitation of unconventional hydrocarbon resources (UHRs), such as shale gas/oil, has been made possible through the application of technologies including horizontal wells completed in multiple hydraulic fracturing stages^1^. However, efficient recovery of UHRs has in part been hampered by a lack of understanding of fluid storage, distribution, displacement and transport processes occurring within the matrix nanopore structure where the majority of the hydrocarbon resource resides^1, 2^. With interest growing in the application of improved oil recovery (IOR) techniques, achieved for example through water and gas injection, and the possibility of permanently storing greenhouse gases (GHGs) in UHRs as an important environmental byproduct of IOR processes^3^, a premium has been placed on understanding fundamental pore-scale physical processes which control injected and produced fluid distribution, displacement and transport mechanisms.

Recent advances in imaging technologies such as focused-ion-beam scanning electron microscopy (FIB-SEM)^4^ have revealed important details regarding the nanopore structure in shales^5^, an important UHR. This pore structure information has been used in the population of pore-scale models to predict important shale reservoir properties affecting fluid storage and flow properties such as porosity and permeability^6^. It is possible that these pore-scale models may even be able to predict important reservoir properties affecting multi-phase flow of gas, water and oil in UHRs, such as relative permeability and capillary pressure. However, while imaging of rock nanopore structure is now routine, imaging of fluid-rock interaction at this scale, necessary for quantifying multi-phase fluid distribution and flow, is not.

**Figure 1:**
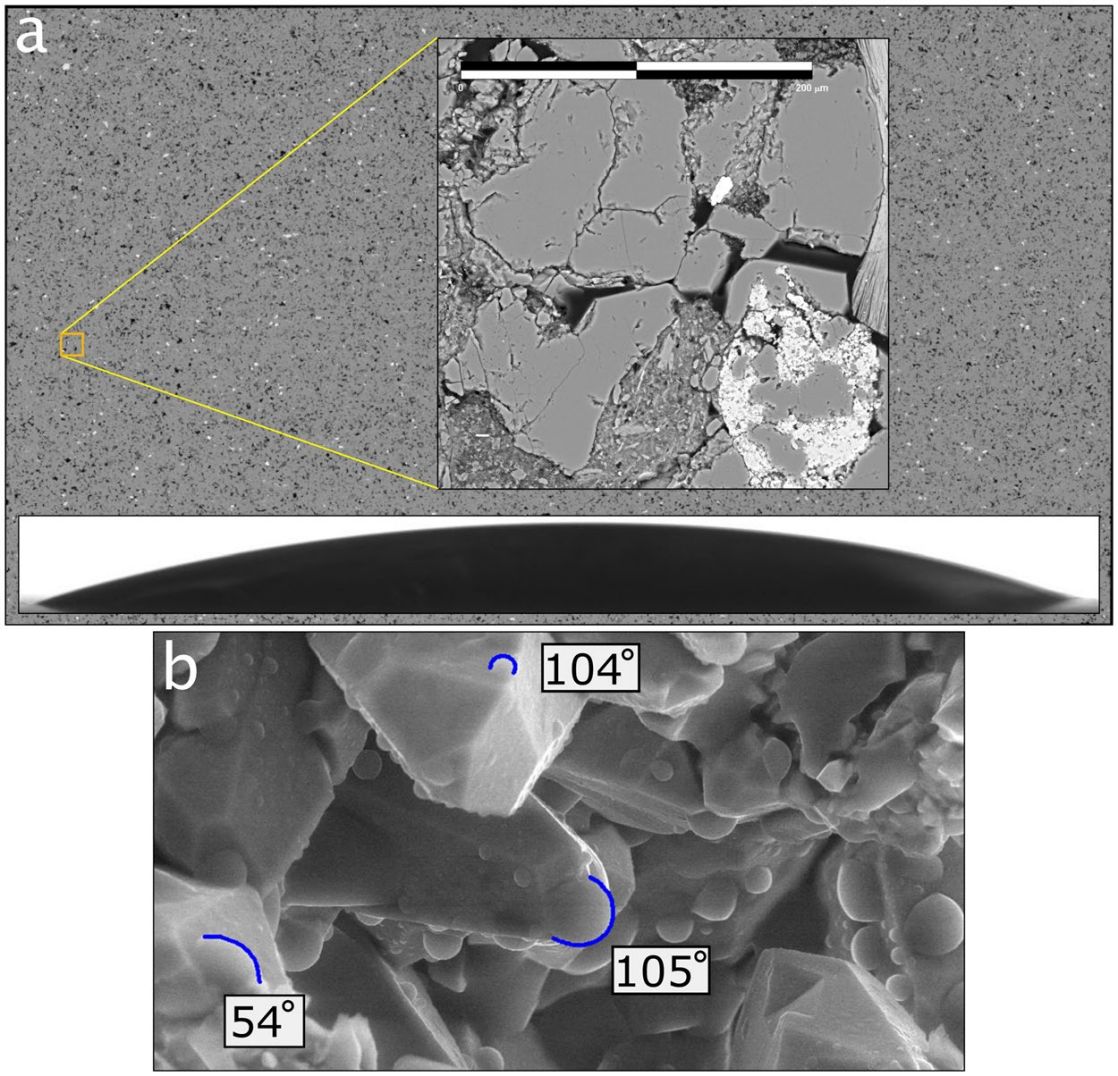
(**a**) Thin-section (2.8 cm × 1.6 cm) of a low-permeability Cardium sample for which back-scattered electron (BSE) images were acquired at high resolution (<250 nm) using an FEI Quanta FEG 250 environmental field emission scanning electron microscope (E-FESEM). The Cardium sample was first impregnated with epoxy, then finely polished and mounted on a slide. A zoom-in of the area outlined with the orange box illustrates the high spatial resolution. Fine-scale (micro- or nano-meter) pore structure information, such as that illustrated in the zoomed image, is often used to populate pore-scale models for permeability and porosity estimation. However, wettability measurements are still routinely performed at the macro-scale (see hypothetical macro-droplet superimposed on the bottom half of the thin section), which cannot be used to model fluid flow at the pore scale. (**b**) Image (collected using E-FESEM) of micro-droplet formation on a Cardium sample during a condensation/evaportation experiment illustrating water contact angle variability at the grain- (micron) scale.

Indeed, characterization of fluid-rock interaction for UHRs is currently limited to the macro (mm) - scale^7^. Routine methods for assessing macro-wettability of fluids on rock surfaces (as determined from contact angle measurements) include the sessile drop and captive bubble techniques^8, 9^, which are commonly used by research/commercial laboratories mainly due to their simplicity. However, these two techniques can provide quite different contact angles depending on the degree of heterogeneity/roughness of the solid surface, the drop (bubble) size, and environmental vibrations^10^. Further, contact angle measurements performed at the macro-scale can only be used to characterize the average wettability of a rock-fluid system. This latter point is illustrated conceptually in Fig. 1a where back-scattered electron (BSE) images of a low-permeability (Cardium Formation) sample were acquired using an SEM^1^. Imaging over a thin-section-scale sample (2.8 cm × 1.6 cm) was performed at high imaging resolutions (<250 nm). The inset image illustrates the fine-scale variation in mineralogy (evident from gray-scale variation) and ultra-fine pore structure – pore structure images of this resolution may be used to populate pore-scale models. However, conventional macro-scale measurements of wettability, illustrated with a conceptual macro-drop placed on the bottom half of the thin section image, would provide an average wettability that cannot be used in modeling fluid flow through the fine pore-structure in contact with mineral grains with variable composition. For example, Fig. 1b is an SEM image of a Cardium Formation sample taken during a condensation/evaporation experiment (see “Results” section) illustrating variability in droplet contact angles at the micro-scale for different mineral grains.

In the present work, quantification of micro-scale variations in wettability and fluid distribution in a low-permeability oil reservoir for use in pore-scale modeling is made possible with an environmental field emission scanning electron microscope (E-FESEM). The studied samples were taken from the Bakken reservoir in Viewfield Saskatchewan, Canada, which is an active low-permeability oil development area^11^. Importantly, in-field piloting of IOR processes is being undertaken in the middle member of the Bakken^12^ (hereafter referred to as “middle Bakken”) and the present work and other reservoir characterization efforts will allow field-scale models to be constructed to evaluate both hydrocarbon recovery efficiency and CO_2_ storage potential. Pore-scale models will first be generated to derive important reservoir properties such as relative permeability and capillary pressure curves^13^, but a key uncertainty to be addressed in the current work is the effect of micro-scale rock compositional heterogeneity on fluid-rock interaction.

A logical order of experimentation with the E-FESEM was used to assess micro-wettability in the mineralogically-heterogeneous middle Bakken tight oil samples. First, live imaging of distilled water condensation/evaporation experiments, due to relative experimental simplicity, was undertaken to provide a base dataset for: 1) testing micro-droplet profile extraction and contact angle-fitting algorithms and 2) assessing the variability in micro-droplet contact angles on sample surfaces. Once the algorithms were vetted, cryogenically frozen samples were then analyzed after sequential imbibition of reservoir oil then synthetic brine to evaluate “native” fluid distribution and contact angles. The combination of SEM imaging and X-ray mapping of the cryogenically frozen samples allowed for both fluid identification and fluid-rock interaction analysis. Finally, live imaging of the injection and subsequent fluid-rock interaction of nanoliters of fluid, using a micro-injection system, was performed. Here live imaging refers to a sequence of time-lapsed images taken at 1 frame per second. These latter experiments, the first of their kind, were used to determine if sessile and dynamic micro-droplet contact angles of arbitrary fluids could be measured.

This work builds on that of previous studies using environmental scanning electron microscopy (ESEM)^14–16^, cryogenic scanning electronic microscopy (cryo-SEM)^17–22^ and other imaging studies^23^ focused on the wetting behavior of micro- (and nano-) droplets on a variety of substrates. As a result, the current advances put forward, particularly in the area of controlled micro-injection of nanoliters of fluid, have broader applicability in various scientific fields where the study of wetting phenomena at a fine scale is necessary.

## Results

### Condensation and evaporation

**Figure 2:**
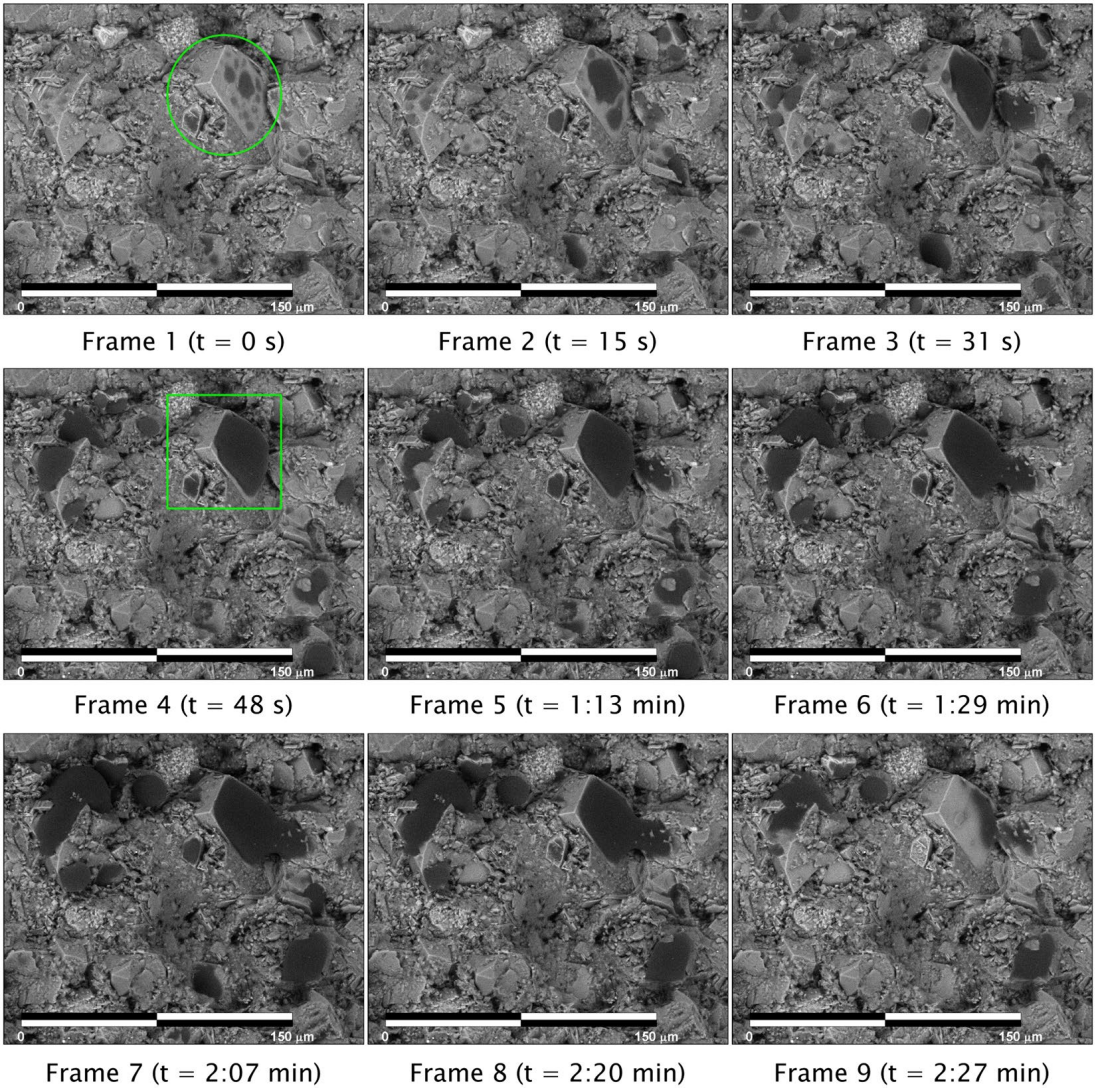
Time-lapse images (compiled over 2:27 minutes) of a condensation/evaporation experiment performed on a middle Bakken sample using the E-FESEM. Micro-droplets of distilled water can be seen forming on the large dolomite grain, annotated by the green circle (Frame 1). As the experiment progresses, the droplets coalesce into a larger micro-droplet (Frames 2–4) which can then be used for contact-angle measurement (green box in Frame 4). The micro-droplet in Frame 4 was chosen for this purpose because, later in the condensation process (Frames 5–6), pinning and the merging of the micro-droplet with a micro-droplet in an offset grain affects the contact-angle measurement. Evaporation occurs during Frames 8–9. The sample temperature was held at around 2.0 °C, and chamber pressure around ~700 Pa. The scale bar is 150 µm.

**Figure 3:**
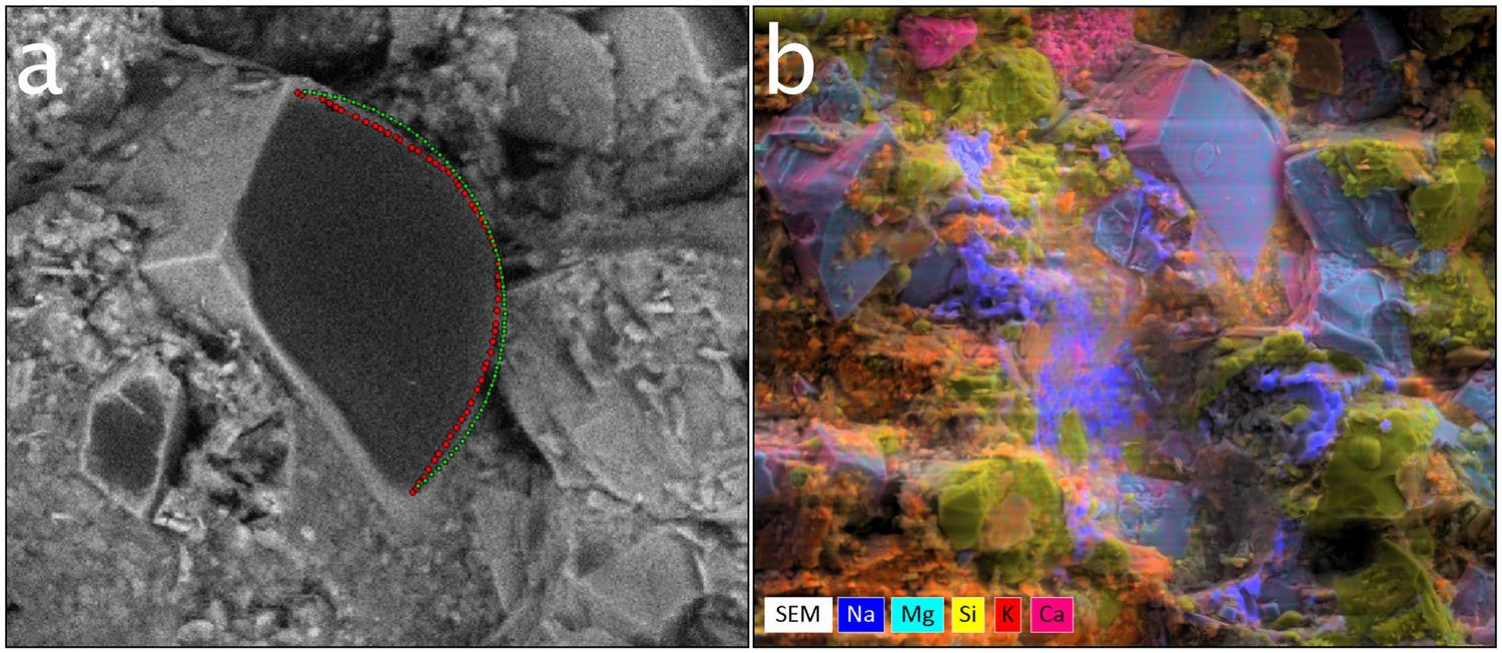
(**a**) A zoom-in of the dolomite crystal in Frame 4 of Fig. 2 illustrating 1) the extracted droplet profile (red dotted line) used for contact-angle assessment and 2) the fit of the parameterized Young-Laplace equation (green dotted line) used to estimate left and right contact angles, and droplet dimensions. The procedure for micro-droplet contact angle assessment is provided in the “Methods” section. The measured drop height and width is 17.1 μm and 48.4 μm, respectively. The right and left contact angles are 69° and 69°, respectively. (**b**) Elemental map (created from X-ray detection and analysis), superimposed on the secondary electron image of Fig. 2. The following minerals are interpreted from the elemental mapping: dolomite (determined from Mg, light blue), quartz (Si, yellow), potassium feldspar (K, red-orange), calcite (Ca, pink) and salt (Na, dark blue).

By using a Peltier stage for heating/cooling the samples, condensation and evaporation of distilled water can be carefully controlled and imaged. As such, the (time-lapse) evolution of sessile droplet formation onto samples can be observed, allowing for the appropriate moment to select droplet profiles for contact angle estimation. This is illustrated in Fig. 2, which provides a time-lapse sequence (2:27 minutes) of a condensation/evaporation experiment for a middle Bakken sample, captured using back-scatter-electron imaging. Focusing attention on the dolomite grain (circled in Frame 1), it can be observed that micro-droplet growth occurs on the grain until Frame 7, after which an evaporation cycle initiates. Frame 4 is selected for droplet profile extraction (Fig. 3a) because the contact angle in later frames is affected by pinning and the micro-droplet wetting and merging with a micro-droplet growing on an offset grain (Frames 5–8). In this example, pinning refers to change in the three-phase contact line during subsequent addition of fluid^24^.

Once the appropriate frame for analysis has been selected, the droplet profile is extracted, and a parameterized version of the Young-Laplace Equation^25, 26^ is fit to the droplet profile (see “Methods" section for procedure) to obtain the contact angles on each side of the droplet (Fig. 3a). The resulting extracted micro-droplet dimensions are 17.1 μm (height) × 48.4 μm (width), with an average (of left and right) contact angle of 69°.

Using this procedure, additional micro-droplet profiles were extracted from the imaged region of the sample shown in Fig. 2. Micro-scale wettability of this sample is variable. An X-ray elemental map (Fig. 3b), overlaid on the secondary-electron image, reveals compositional variability of the sample, which in turn can be used to assess the compositional controls on wettability.

**Figure 4:**
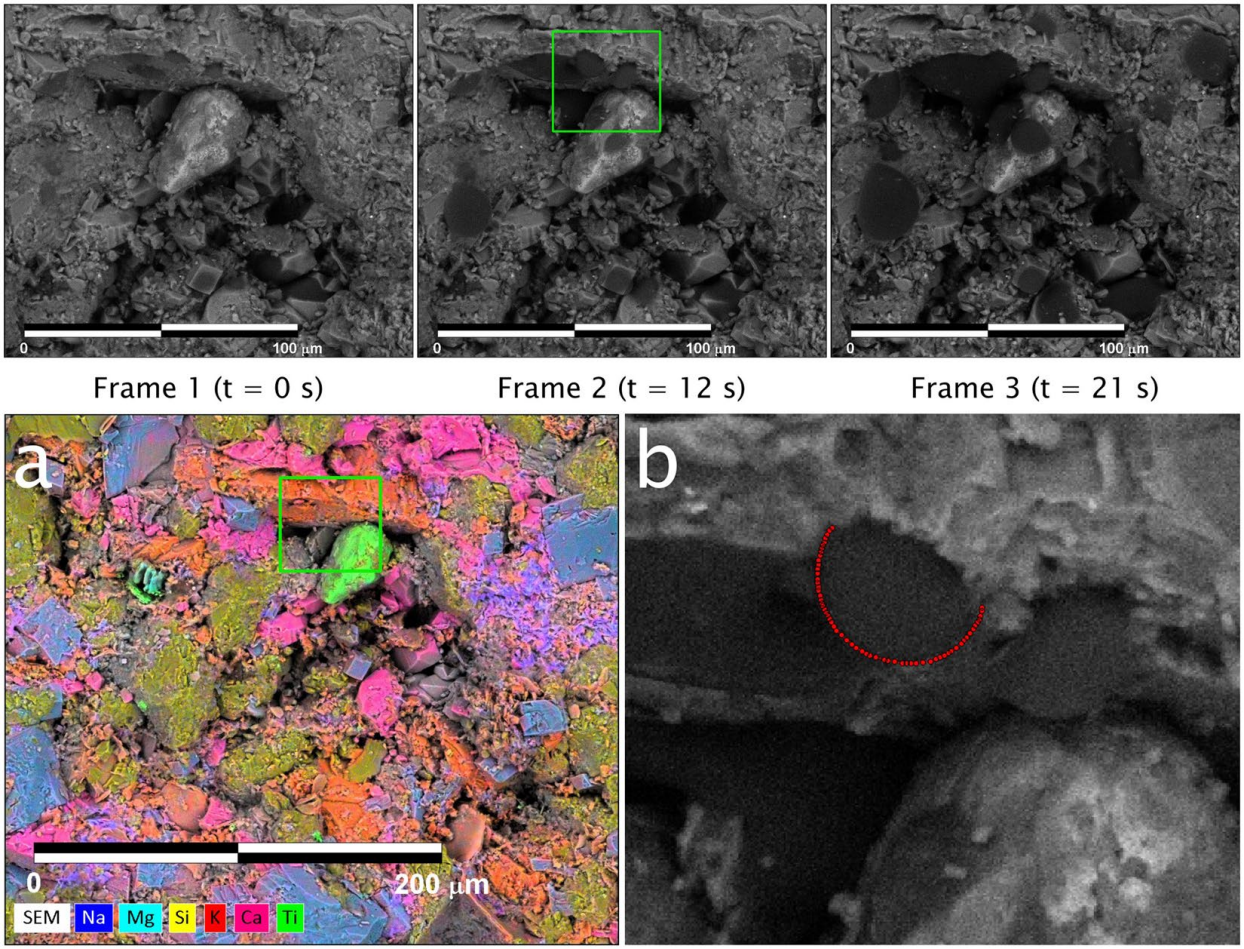
The first 3 frames are time-lapse images (compiled over 21 seconds) of a condensation/evaporation experiment performed on a second middle Bakken sample using the E-FESEM. Water condensation is seen to occur on a rutile grain (tear-drop shaped grain appearing in the lower half of the green box in Frame 2 and on a potassium feldspar grain whose edge can be seen in the upper half of the green box). The sample temperature was held at around 2.1 °C, and chamber pressure around ~700 Pa. The scale bar is 100 µm. (**a**) Mineral identification through the use of elemental maps. Color coding for elements/minerals is the same as for Fig. 3b with the addition of titanium (light green) corresponding to rutile. (**b**) Zoom in of the green box in (**a**) showing an extracted droplet profile (red dotted line) for a micro-droplet condensed onto the surface of the potassium feldspar grain. The measured micro-droplet height and width is 6.8 µm and 12.2 µm, respectively. The calculated right and left contact angles are 94° and 97°, respectively.

An additional time-lapse example (21 seconds) is provided in Fig. 4. This sample is compositionally distinct from that shown in Fig. 3b, due in part to the presence of rutile (light green on elemental map in Fig. 4a) and potassium feldspar (orange on elemental map), providing an opportunity to further evaluate the relationship between composition and wettability. Micro-droplets such as those condensing on the potassium feldspar grain adjacent to the rutile grain (highlighted in green box, Frame 2 of Fig. 4) can be analyzed using profile extraction and fitting of the Young-Laplace Equation (Fig. 4b). The resulting extracted micro-droplet dimensions are 6.8 μm (height) × 12.2 μm (width), and an average contact angle of 95.5°.

### Cryogenic

Imaging and X-ray mapping of samples after cryogenically freezing them allows the distribution of native fluids, and native fluid contact angles, to be determined, if preserved samples are available. This same technology can be used to evaluate the distribution of fluids and their contact angles after they are imbibed or injected into the sample. An example of the latter is studied herein.

**Figure 5:**
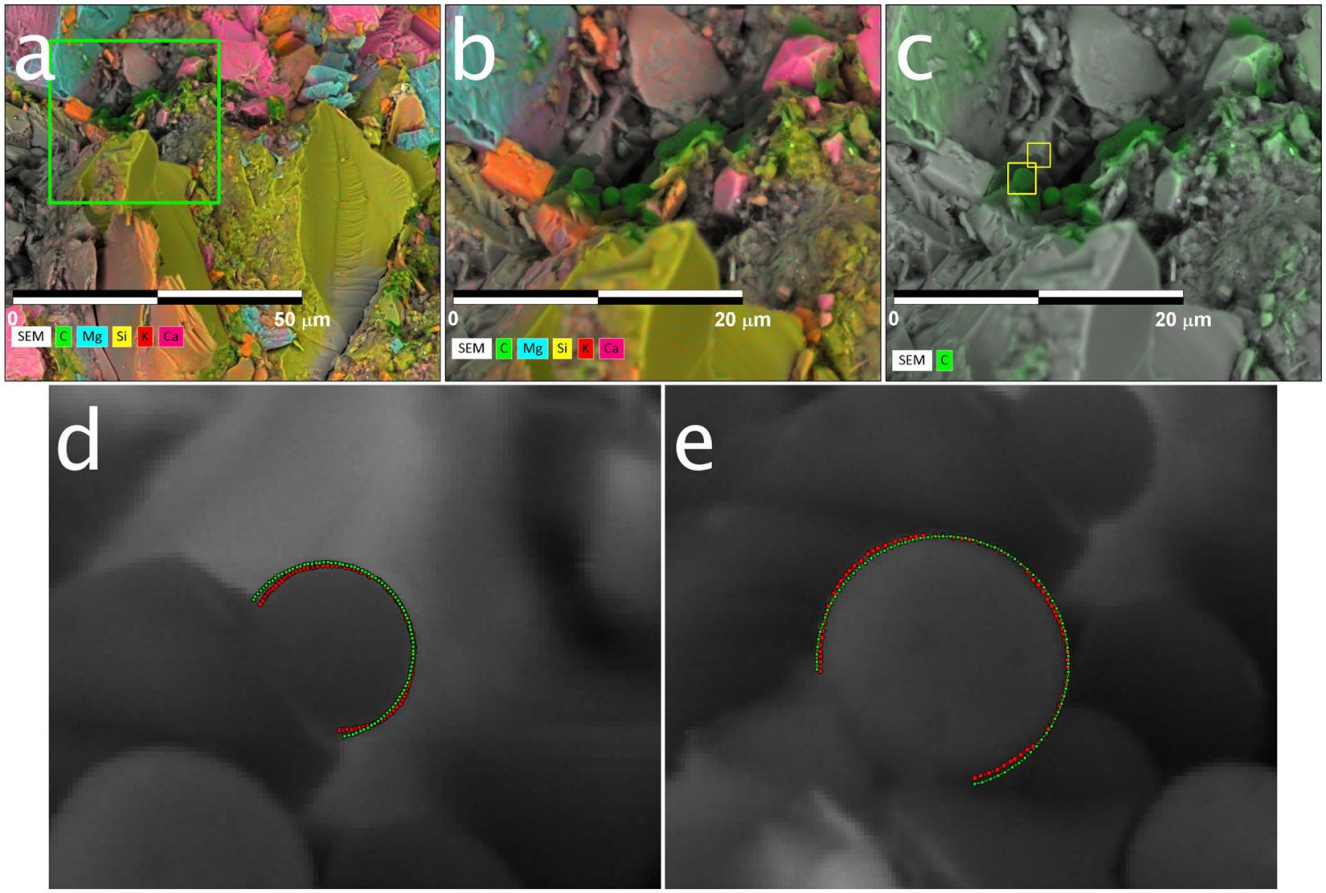
(**a**–**c**) Elemental maps superimposed on a back-scatter electron image of the cryogenically-frozen middle Bakken sample after it had been submersed in formation oil. The elemental map in (**a**) is zoomed in (**b**) (green box, 30 µm wide) to reveal oil micro-droplets (green) in the intergranular space. In (**c**), only the element carbon is shown, emphasizing the location of the oil droplets in the intergranular space. (**d**,**e**) The oil droplets highlighted in the yellow boxes in (**c**) are zoomed in to illustrate the fitting of the parameterized Young-Laplace Equation (green dotted line) to the extracted droplet profile (red dotted line). The micro-droplet in (**d**) has a measured drop height and width of 671 nm and of 871 nm, respectively, and calculated right and left contact angles of 113° and 109°, respectively. The micro-droplet in (**e**) has a measured drop height and width of 1.3 µm and of 1.2 µm, respectively, and calculated right and left contact angles of 128° and 130°, respectively.

A middle Bakken sample was immersed in formation oil (obtained from the wellsite) for six weeks, allowing oil to be imbibed into the sample, and was subsequently cryogenically frozen, broken to expose a fresh surface, and imaged (Fig. 5). X-ray (element) mapping (Fig. 5a) again reveals the sample compositional heterogeneity; further, the location of imbibed oil in the sample can be identified using the carbon element map and the appearance of micron-sized oil droplets (Fig. 5b,c). Two oil micro-droplets are analyzed - one droplet has dimensions of 0.67 μm (height) × 0.87 μm (width) and an average contact angle of 111°, while the other has dimensions of 1.3 μm (height) × 1.2 μm (width) and an average contact angle of 129°. Because of the irregularity of the sample surface, it is difficult to assess what mineral(s) surface the oil droplets are adhering to; the immediate region surrounding the micro-droplets appears to be rich in quartz, consistent with the non-oil-wet nature of the surface.

**Figure 6:**
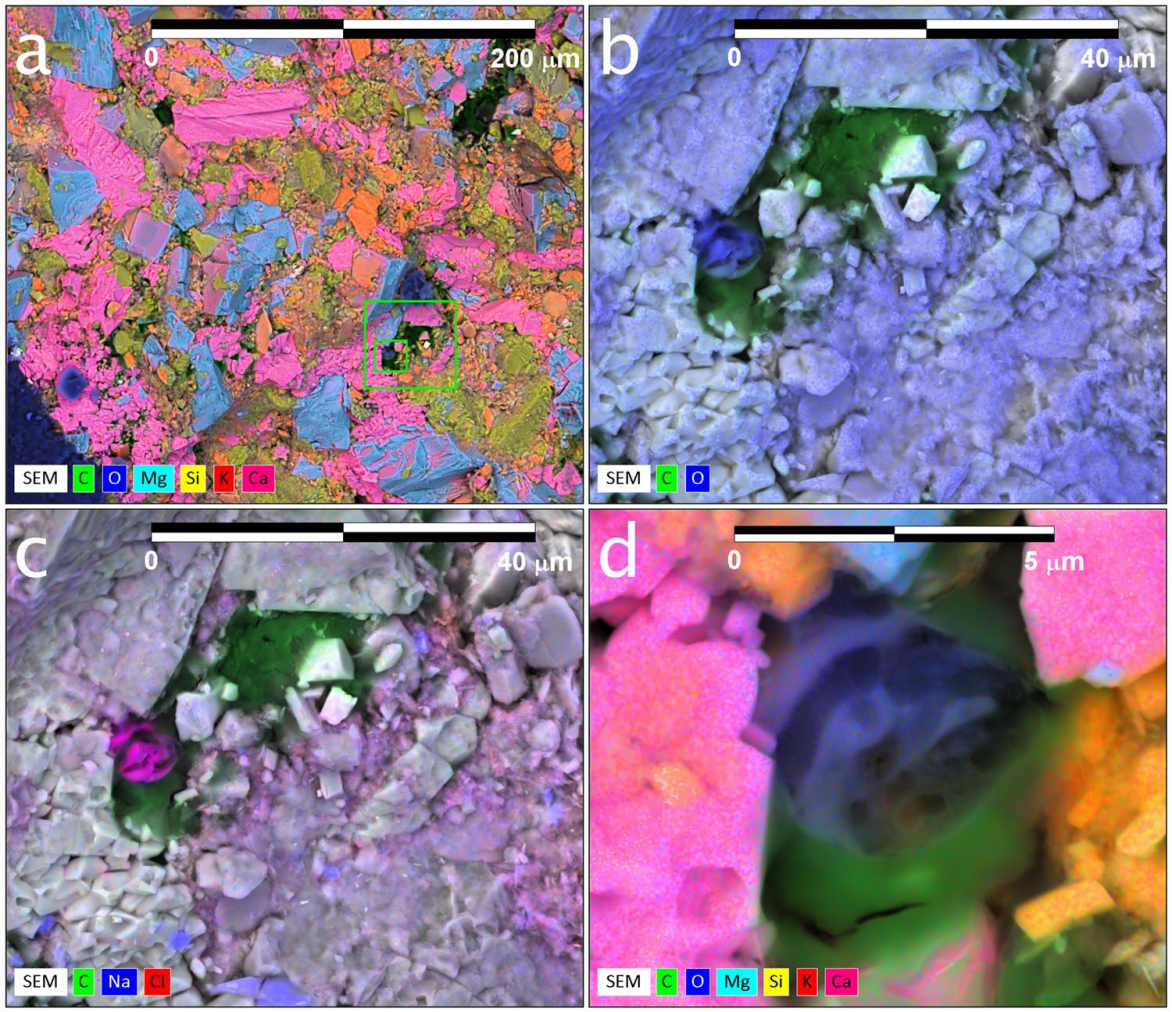
(**a**) Elemental map (300 µm × 300 µm) superimposed on a back-scatter electron image of the cryogenically-frozen Bakken sample after it was first submersed in formation oil, then in synthetic brine. (**b**) Zoom in of larger green square in (**a**) illustrating elemental map of carbon and oxygen only, and location of oil (green) and synthetic brine (blue) in intergranular space. (**c**) Same image as (**b**), but with the elements of sodium and chloride added instead of oxygen to demonstrate that the identified water in the intergranular space is synthetic brine. (**d**) Zoom in of smaller green square in (**a**) to illustrate the distribution of oil (green) and synthetic brine (blue) in the intergranular space.

The same middle Bakken sample was then immersed in synthetic brine for 3^1/2^ weeks, and the procedure repeated. Unfortunately, imbibition of the synthetic brine was limited to locations near the sample edges. The elemental map (Fig. 6a) again reveals mineralogical heterogeneity; after zooming (see green squares in Fig. 6a), synthetic brine and oil distribution in the inter-granular space can be observed, although no contact micro-droplet profiles are available for analysis (Fig. 6b,d). Sodium and chlorine elemental maps (Fig. 6c) confirm that the entrained water is synthetic brine.

### Micro-injection

**Figure 7:**
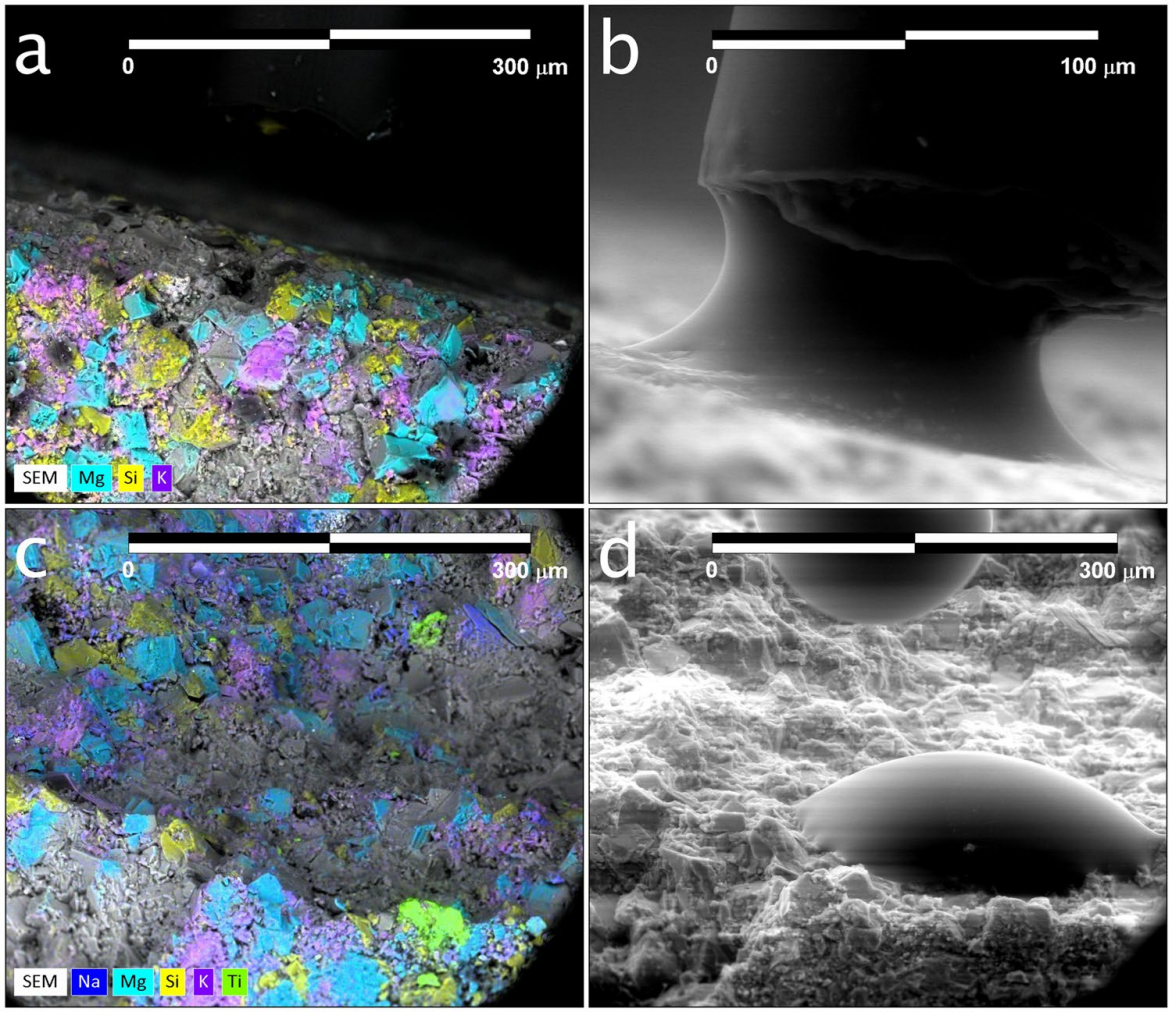
(**a**) Elemental map of the edge of a middle Bakken sample (Sample 1A) used for micro-injection analysis. This sample orientation is nearly orthogonal to the imaging plane, offering an ideal view of micro-droplet formation. (**b**) A secondary-electron image of water injected out of the silica tube (10 µm ID, 150 µm OD) onto Sample 1A during a micro-injection experiment. This image is a still taken from a sequence of time-lapse images used for evaluation of advancing and receding contact angles (not shown). (**c**) Elemental map taken of a less-ideally oriented Bakken sample (Sample 1B) used for micro-injection analysis. (**d**) A secondary electron image of sessile droplet formation on Sample 1B during a micro-injection experiment. This image is a still taken from a sequence of time-lapse images (Fig. 8) used for evaluation of micro-droplet contact angles during water imbibition.

**Figure 8:**
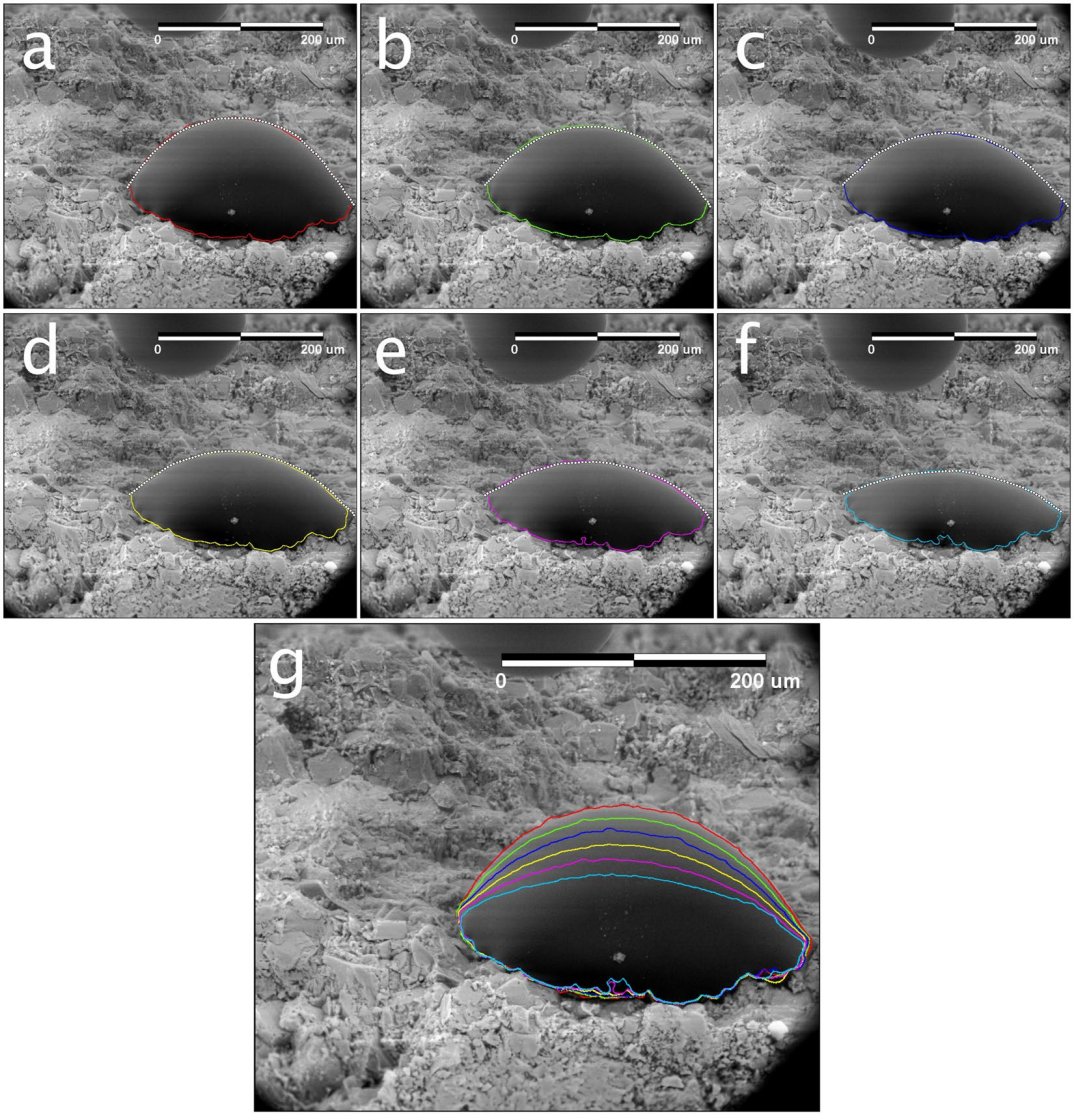
(**a**–**f**) Time-lapse images (combined secondary and back-scattered electron) of micro-droplet imbibition into Sample 1B. The colored outlines in each frame illustrate the extracted droplet profile, while the white dotted lines represent the fitted, parameterized Young-Laplace Equation to the outer surface of the micro-droplet. The frame time is one second. (**a)** Red: the calculated left and right contact angles are 65° and 51°, respectively. (**b**) Green: the calculated left and right contact angles are 56° and 46°, respectively. (**c**) Dark blue: the calculated left and right contact angles are 51° and 38°, respectively. (**d**) Yellow: the calculated left and right contact angles are 40° and 30°, respectively. (**e**) Purple: the calculated left and right contact angles are 32° and 26°, respectively. (**f**) Light blue: the calculated left and right contact angles are 23° and 21°, respectively. (**g**) Summary image illustrating micro-droplet profile changes during imbibition. The scale bars are 200 µm.

The micro-injection procedure represents the most important and innovative advancement in micro-wettability determination presented herein. Two middle Bakken samples are examined to demonstrate its utility. One of these samples (Sample 1A), is oriented such that the surface on which the fluid is placed is nearly orthogonal with the imaging plane (Fig. 7a), while the other has a significant amount of surface topography and is less ideally oriented (Fig. 7c). Nanoliters of fluid were injected on to the surface of each sample through the micro-injection system – advancing and receding contact angles were first measured while the fluid was dragged back and forth across the surface of the sample (Fig. 7b). Sessile droplet contact angles were then measured when the micro - capillary tube was pulled away from the surface (Fig. 7d) as the droplet was imbibing into the sample. A montage of time-lapse images associated with the latter process is provided in Fig. 8a through Fig. 8f. For each image, droplet profiles were first extracted (colored outlines), and the parameterized Young-Laplace Equation fit to the upper surface of the micro-droplet (white dots). Figure 8g provides a summary of the extracted profiles over time. The droplet profile change can be used to estimate imbibition rate, which in turn can be used to understand the time-scale of hydraulic-fracture fluid imbibition into unconventional reservoirs – this will be the subject of future work.

## Discussion

Each of the methods described above for evaluating micro-scale wettability have strengths and weaknesses. Further, the contact angle/interfacial tension measurements obtained using each method are significantly affected by micro-droplet profile orientation and surface roughness. These topics are explored further in this section.

Condensation/evaporation experiments achieved through cooling/heating the sample using the Peltier stage, and consequent precise control of water saturation pressure, are the simplest to perform. These experiments have been carried out on a variety of rock types of variable composition, fabric and texture, and the technology is well-established. Sessile droplet formation on discrete mineral surfaces (Fig. 1) is a consistent result of the experiments and contact angle measurement is repeatable. Indeed, the contact angle extraction methodology developed herein was first perfected using this simple experimental procedure. One problem encountered with these experiments is related to the concentration of hygroscopic minerals such as salts, which may be a product of formation brine evaporation. When hygroscopic minerals are prevalent in the sample, they can cause condensation clustering, restricting condensation to areas of higher concentration of salts, and thus redirecting condensation away from areas of interest. The principle disadvantage of the condensation/evaporation methodology, however, is that it is restricted to the study of distilled water wettability (condensed from water vapor in the sample chamber). While such studies may be useful for evaluating sensitivity of the rock sample to fresh water, which is of importance if fresh water-based fluids are used for hydraulic fracturing, it will not allow wettability to brines or native fluids to be determined.

Cryogenic experiments are better suited for native fluid wettability evaluation. It is possible to obtain preserved cores from wells where native fluids are retained; cryogenically freezing sub-samples of these cores, combined with the electron imaging and X-ray mapping methodology described herein, may therefore be used to evaluate native fluid (brines and hydrocarbons) distribution and wettability (if micro-droplets are observed), and their relationship to rock composition. However, preserved cores, which require specialized procedures for downhole retrieval, transfer from wellsite to lab, and handling, are not commonly collected. As illustrated in this study, fluids may be imbibed or reinjected into the sample to simulate drainage (decrease in wetting fluid saturation) and imbibition (increase in wetting fluid saturation) processes, but if the samples are not properly preserved, it is nearly impossible to simulate *in-situ* fluid distribution. A further disadvantage of the cryogenic experiments using the fluid imbibition approach in these samples is that micro-droplet formation is not controllable and is more random than with condensation/evaporation and micro-injection experiments. However, use of preserved core samples, where native fluids are retained in the sample, may help rectify this.

Micro-injection experiments offer significant advantages over the other micro-wettability methods used in this study. A principal advantage is that a wider variety of fluids may be selected for examination including aqueous fluids, brines, non-aqueous fluids, and very importantly, fluids used during drilling and hydraulic fracturing operations. The experiments can be specifically designed to evaluate the susceptibility of formation samples (and specific regions of the samples, depending on mineral distribution) to damage by fluids introduced to the formation during drilling and hydraulic fracture stimulation. In principal, fluid wettability alteration due to fluid chemistry changes, or addition of nanoparticles, may be studied through live imaging during micro-injection – this opens up a new avenue for fracturing fluid design, for example. The micro-injection system developed herein can be used to inject as little as a few nanoliters of fluid – this high-precision conveyance system combined with nm-scale position control of the micro-capillary afforded by the micro-manipulator, allows small regions (e.g. mineral grains) to be effectively targeted for sessile droplet contact angle measurement. This control is not afforded by the other techniques. Further, once the fluid is conveyed to the sample, and while still attached to the micro-capillary, it may be dragged across the sample surface to perform advancing/receding contact angle measurements. However, as with the other techniques, only grain surfaces that are suitably oriented for imaging may be used to provide contact angle measurements.

In order to quantify the errors in contact angle estimation associated with sub-optimally oriented samples, a series of simulations were performed whereby contact angles were extracted from micro-droplets intersected by planes at varying angles (0–60°). Using the procedure for contact angle estimation described in the “Methods” section, errors in droplet contact angle, as well as droplet height, diameter and interfacial tension were quantified for each of the offset angles. As a general conclusion, these errors become greater with a) an increase in droplet contact angle and b) degree of offset. Errors are therefore expected to be greater for non-wetting phases, and for contact angles observed at oblique angles. A priority for future work is therefore to improve contact angle imaging orientations, and reduce surface topography (without altering wettability). For samples that are not ion-milled, we are currently exploring techniques to “correct” for the effects of surface roughness, including mapping the topography using stereo-pair images or optical profilometry.

The current experimental setup allows measurement of contact angles at low temperature (typically ~2 °C) under vacuum (~650–800 Pa). To determine the relevance of the wettability experiments with respect to *in-situ* reservoir conditions, previous studies that investigated the effects of temperature and pressure on contact angles were considered^27–31^. The observed trends depended on the conditions of the system that was being researched. In general, it has been found that contact angles are not very sensitive to pressure, but temperature has a more significant effect. For example, Najafi-Marghmaleki *et al*.^31^ studied four different water-wet carbonate oil reservoirs, and noted that varying pressure had no significant effect on the measured contact angle of those reservoirs. However, an increase in temperature changed the wettability of some reservoirs from water-wet to intermediate-wet. The Peltier stage used in the current work has a temperature range of −25 °C to 60 °C; however the temperature range available to realistically conduct these experiments is much more severely limited by the range of pressures available in the E-FESEM for imaging in a humid sample chamber environment. Future work, therefore, will include assessing the effect of temperature on contact angle in macro-wettability experiments on single-mineral systems. The resulting trends can then be used to extrapolate the micro-wettability results to reservoir conditions.

Despite possible errors in contact angle estimation due to sub-optimally oriented samples, the micro-wettability estimation procedures outlined in this work provide a significant advance in the quantification of contact angle variability at the micro-scale. Mohammadmoradi and Kantzas^32^ note that contact angle heterogeneity can be caused by mineral complexity and wettability alteration due to “aging”. Those authors go on to illustrate the important control that contact angle heterogeneity has on micro-scale displacement processes (drainage and imbibition). Those authors^33^ and others^34, 35^ further illustrate the important control that wettability changes at the micro-scale can have on electrical conductivity (which in turn impacts petrophysical evaluations of water saturation, for example) and thermal conductivity (which is used in the evaluation of thermal recovery operations in heavy oil reservoirs). While wettability heterogeneity due to aging is not addressed in this work, the procedures developed herein can be used to quantify micro-wettability with changes in mineralogy (as evaluated using X-ray mapping), allowing for wettability to be mapped across the surface. With this information in hand, more accurate assessments of key properties affecting multi-phase flow (e.g. capillary pressure and relative permeability) are now possible with the assistance of pore-scale modeling^13, 36, 37^, which in turn will result in improved evaluation of primary and enhanced recovery processes in unconventional reservoirs. However, wettability is typically measured at the macro-scale, with the resulting contact angles used for populating the pore-scale models. Future work will include studying the results of comparing contact angle measurements made at the macro-scale with those made at the micro-scale (as outlined above), to understand the significant errors that may occur in simulating fluid displacement processes, fluid saturation distributions, capillary pressure and relative permeability curves using pore-scale models if the micro-scale variations in wettability are not taken into account.

## Conclusions

Three approaches (condensation and evaporation, cryogenic freezing, and micro-injection) were evaluated for quantifying micro-wettability in samples from low-permeability oil reservoirs. The following primary conclusions can be drawn from this work:

1. Of the three methods that were evaluated for quantifying micro-wettability, micro-injection showed the most promise because of the ability to control the location of where the fluid was placed on the sample, observe receding and advancing contact angles, and observe the imbibition rate.
2. The condensation and evaporation experiments were successful in establishing the wettability of mineral crystals within tight oil samples to distilled water. The measured contact angle for a dolomite crystal was 68° while the measured contact angle for a potassium feldspar grain was approximately 95°, with some variation between the right and left contact angles.
3. The cryogenic experiments were successful in imaging oil imbibed into a tight oil sample as well as imaging the oil/brine interface. Measured contact angles for cryogenically frozen oil ranged from 109° to 130°.
4. Error in contact angle estimation increases as the droplets become less wetting (higher contact angles) and as the degree of offset increases.

## Methods

### Instrumentation

Micro-scale variation in wettability and fluid distribution was investigated using an FEI Quanta FEG 250 environmental field emission scanning electron microscope (E-FESEM). This instrument is capable of acquiring secondary electron (SE) and back-scatter electron (BSE) images at nm-scale resolution using a variety of detectors. Experiments can be performed under a range of vacuum conditions (pressures from high vacuum up to 4000 Pa) in a variety of gaseous environments (e.g. high vacuum, air, water vapor, nitrogen, helium). The system is further equipped with FEI Maps software, which allows for the seamless, tiled imaging of arbitrarily large areas at high resolution (see Fig. 1 for example), restricted only by the practical limitations of maximum sample size, data storage capacity, and acquisition time.

A Bruker Quantax EDS system is used for X-ray detection and analysis. The system is comprised of a Quantax 5030 Silicon Drift Detector and associated data acquisition electronics, and a computer running the Bruker Esprit software. Importantly, the system software allows for X-ray element maps to be superimposed on electron images and used to relate mineralogy to wettability.

A rich assortment of auxiliary equipment has made the three methods of micro-wettability determination (condensation/evaporation, cryogenic, and micro-injection) possible. A Peltier heating/cooling stage allows the sample temperature to be controlled in the range of approximately −25 °C to 60 °C. Precise temperature control of the sample at and around the dew point enables the condensation/evaporation experiments, the dew point being pre-determined by carefully controlling the pressure of water vapor in the sample chamber. A Gatan Alto 2500 Cryo-Preparation and Transfer system, and associated Cryo-Stage module, was used for cryogenic experiments. This system allows for rapid freezing and cryofixation of beam- or vacuum-sensitive samples and subsequent cryo-fracture preparation under vacuum. Once the sample is fractured to expose a fresh surface in the Cryo-Preparation chamber, it is transferred to the SEM Cryo-Stage module for examination while maintaining sample temperatures as low as −190 °C. A Kleindeik MM3A-EM micro-manipulator with attached Kleindeik MIS-EM micro-injection valve was used for micro-injection experiments, allowing for the injection of arbitrary fluids onto the sample under examination. To enable the injection of nanoliter volumes of fluid necessary for micro-wettability experiments, a custom injection nozzle consisting of silica tube (10 µm ID, 150 µm OD) coated with polyamide (to prevent water from wicking up the side of the tube) was mated to the injection valve using a Teflon friction fit coupling. For the micro-injection experiments, the Peltier stage with sample, micro-manipulator, and micro-injector are inserted into the E-FESEM chamber. The Peltier stage temperature is controlled via software, while the micro-manipulator and micro-injection valve are controlled via dedicated hardware supplied by Kliendeik. The fluid used for micro-injection is held in a syringe external to the sample chamber and conveyed through a plastic capillary and vacuum feed-through into the sample chamber and on to the injection valve. The volume of injected fluid is determined by the length of time the micro-injection valve is held open and is controlled by external electronics.

### Samples and Sample Preparation

Samples used for micro-wettability evaluation in this study were sub-sampled from core plugs, which in turn were extracted from the middle member of the Devonian- to Mississippian-aged Bakken Formation of the Williston Basin in the Viewfield area of Saskatchewan, Canada^38^. The middle member is comprised primarily of dolomite-cemented siltstones that are actively being developed as a low-permeability, light oil resource^39^. The core plug samples were taken from the middle member of the Bakken primarily for the purpose of characterizing porosity, permeability, and mechanical property trends in support of reservoir simulation studies of enhanced oil recovery in the Viewfield area. The current study was intended to extend the previous middle Bakken characterization work by our research group^40, 41^ to include wettability/micro-wettability characterization.

For condensation/evaporation experiments, a sample wafer was cut from a core plug, and a small fragment of this wafer was broken off with tile nippers. The sample fragment was then mounted into an aluminum sample cup using a thermally - conductive epoxy. Once the epoxy was cured, excess material from the fragment was broken off to expose a fresh surface for imaging. The sample cup was then fitted to the Peltier heating/cooling stage. For analysis, sample temperatures were held between 1.9 and 2.1 °C, and SEM chamber pressures ranged between 650 and 800 Pa. Condensation/evaporation of water vapor was controlled by varying sample temperature with the Peltier stage.

For cryogenic experiments, a sample fragment was again extracted from the wafer. The sample fragment was submerged in formation oil (obtained at a Viewfield Bakken well location by the operator), and allowed to imbibe oil for 6 weeks (42 days). After oil imbibition, some fragments of the sample were examined using the procedure described below, while other fragments were subjected to 3½ weeks (25 days) of spontaneous imbibition of synthetic brine prepared to match as closely as possible a formation water analysis from a nearby offsetting well.

The following steps were used for analysis of these samples:

1. The sample was placed into an aluminum sample holder with “cryo glue”: a viscous fluid at room temperature consisting of Polyvinyl Alcohol, Carbowax and water, that serves as an adhesive to bond the sample to the sample holder at cryogenic temperatures.
2. The sample/sample holder was submerged in a bath of nitrogen slush until reaching thermal equilibrium.
3. The frozen sample was transferred under vacuum to the Cryo-Preparation chamber.
4. In the preparation chamber, the sample was broken to expose a fresh surface using the point of a #10 scalpel blade on a wobble stick manipulated from outside the preparation chamber.
5. The sample was then transferred into the E-FESEM chamber for electron imaging and X-ray mapping to reveal mineralogy and fluid type and distribution in the pore system of the sample.

The synthetic brine was generated as follows:

1. The masses of various salts required to match the ionic concentrations in 1 L of formation brine were first calculated.
2. Bicarbonate and sulphate were introduced as NaHCO_3_ and CaSO_4_, respectively, while Na, K, Ca, and Mg were added as chlorides. A trace concentration of iron in the formation brine was ignored.
3. Deionized water was then added to approximately the 900 mL level, and the flask agitated until all salts were dissolved. Once dissolution was complete, the flask was topped up to the 1 L level with additional deionized water.

For micro-injection experiments, sample preparation was very similar to the condensation/evaporation experiments described above; however, a specially-made sample holder was used in order to orient the sample vertically so that the sample surface was orthogonal to the silica micro-injection tube, and parallel to the microscope’s axis of observation (Fig. 7). This modification allowed the contact angles to be evaluated more confidently.

### Contact Angle Evaluation

Contact angles were obtained by fitting a parameterized version of the Young-Laplace Equation to extracted micro-droplet profiles. The Young-Laplace Equation relates the pressure difference across a curved interface^42^ as follows:

1 $$\gamma (\frac{1}{{R}_{1}}+\frac{1}{{R}_{2}})=\Delta p$$

Where Δ*p* is the pressure difference across the homogeneous fluid interface, *γ* is the interfacial tension, and *R*_1_ and *R*_2_ are the principal radii of curvature at point *P* on the surface of the micro-droplet. Assuming an axisymmetric interface, Equation 1 can be expanded into a system of ordinary differential equations as a function of arc length *s* ^26^:

2 $$\frac{dx}{ds}=\,\cos \,\theta ,$$

3 $$\frac{dz}{ds}=\,\sin \,\theta ,$$

4 $$\frac{d\theta }{ds}=2b+cz-\frac{\sin \,\theta }{x},$$

5 $$x(0)=z(0)=\theta (0)=0$$

where *b* is the curvature of the interface at the origin, and *b* and $c=\frac{\Delta \rho }{\gamma }g$ are fitting parameters.

The following procedure is used to evaluate micro-droplet contact angles:

1. The micro-droplet profile is first extracted from the SEM image with user-guided software (red dotted line in Fig. 3, for example).
2. Once the profile has been extracted, it is rotated to be horizontal in image coordinates (pixels).
3. The profile is converted to physical coordinates (μm).
4. A fourth order Runge-Kutta method, with truncation error to control the solution, is used to numerically solve and fit the set of parameterized equations to the micro-droplet profile in physical coordinates. From the solution, the left and right contact angles are determined.
5. For comparison with the imaged micro-droplet, the model fitted profile is converted back to image coordinates and rotated back to the original droplet profile orientation (see green dotted line in Fig. 3, for example).

As noted in the “Discussion” section, sub-optimally-oriented samples can cause significant errors in contact angle and interfacial tension determination using this procedure.
